# Recruitment and Baseline Characteristics for the Lifestyle, Exercise and Diet (LEAD) 2.0 Trial: A 6‐month randomized controlled trial of virtual exercise and nutrition interventions in older adults with subjective cognitive decline

**DOI:** 10.1002/alz70860_106557

**Published:** 2025-12-23

**Authors:** Bobby Neudorf, Noah Koblinsky, Krista Power, Malcolm Binns, Alexandra J. Fiocco, Shlomit Rotenberg, Susan Marzolini, Paul I. Oh, Jane Thornton, Fatim Ajwani, Kylie Sullivan, Stéphanie Chevalier, Caryl Russell, Guylaine Ferland, Nicole D. Anderson, Laura E. Middleton

**Affiliations:** ^1^ University of Waterloo, Waterloo, ON, Canada; ^2^ Baycrest Academy for Research and Education, Toronto, ON, Canada; ^3^ University of Ottawa, Ottawa, ON, Canada; ^4^ Toronto Metropolitan University, Toronto, ON, Canada; ^5^ University of Toronto, Toronto, ON, Canada; ^6^ Toronto Rehabilitation Institute, University Health Network, Toronto, ON, Canada; ^7^ University of Western Ontario, London, ON, Canada; ^8^ Toronto Rehabilitation Institute, Toronto, ON, Canada; ^9^ McGill University and Research Institute of the McGill University Health Centre, Montreal, QC, Canada; ^10^ Université de Montréal, Montréal, QC, Canada; ^11^ Schlegel‐UW Research Institute for Aging, Waterloo, ON, Canada

## Abstract

**Background:**

The aim of the LEAD 2.0 Trial is to understand whether a 6‐month online exercise and diet intervention is feasible among older adults at risk for dementia. Here we describe preliminary data on recruitment feasibility and baseline characteristics of LEAD 2.0 participants.

**Method:**

The LEAD 2.0 Trial is a 6‐month factorial, randomized controlled trial among older adults with subjective cognitive decline (SCD). Feasibility will be evaluated based on recruitment (target: 9 participants/month), adherence (target: 75% of sessions attended), and retention (target: 80% of participants complete 6‐month assessment). The exercise intervention included two virtual, group sessions and one independent, pre‐recorded video session each week. The diet intervention included one weekly session of healthy diet counselling with goal setting. Stretch and brain education served as time/frequency/social matched controls for exercise and diet counselling interventions respectively. Assessments at baseline, 6 months, and 12 months were also conducted virtually.

**Result:**

A total of 574 participants were screened from July 2023 to November 2024, with the greatest proportion responding through targeted e‐blasts through ZoomerMedia Limited (46.9%), research participant databases (12.2%), and participant referrals (8.4%). Of those sources, the highest portion of eligible individuals came from research participant databases (45.7%), promotion through local/provincial/national organizations (37.5%), and participant referrals (31.3%). Of 574 individuals screened, 236 passed lifestyle screening, 167 passed cognitive screening, and 146 were randomized (9.1 recruited per month). Eligibility was expanded from executive SCD to all SCD after one month of recruitment. Average age of participants was 72.5 ± 5.2 years, 80.8% were female, 85.6% were White, 88.4% had a college/university degree or greater, 61.6% were married, and 79.5% were retired.

**Conclusion:**

Recruitment to the LEAD 2.0 Trial occurred at target rates using a broad range of recruitment avenues. Targeted e‐blasts produced the largest number of participants screened, while research participant databases produced the greatest recruitment yield. Men, people of minority ethno‐cultural groups, and people with low education remain under‐represented.

